# Temperature and Photoperiodic Response of Diapause Induction in *Anastatus japonicus*, an Egg Parasitoid of Stink Bugs

**DOI:** 10.3390/insects12100872

**Published:** 2021-09-26

**Authors:** Can Zhao, Yi Guo, Zixin Liu, Yue Xia, Yuyan Li, Ziwei Song, Baoxin Zhang, Dunsong Li

**Affiliations:** 1 Guangdong Provincial Key Laboratory of High Technology for Plant Protection, Plant Protection Research Institute, Guangdong Academy of Agricultural Sciences, Guangzhou 510640, China; zhaocan@gdaas.cn (C.Z.); guoyi@gdaas.cn (Y.G.); yue10dp2@126.com (Y.X.); songzw@gdaas.cn (Z.S.); zhangbx@gdppri.cn (B.Z.); 2 Laboratory of Bio-Pesticide Creation and Application of Guangdong Province, Department of Entomology, College of Plant Protection, South China Agricultural University, Guangzhou 510642, China; 15581000722@163.com; 3 Key Laboratory of Integrated Pest Management in Crops, Ministry of Agriculture, Institute of Plant Protection, Chinese Academy of Agricultural Sciences, Beijing 100193, China; liyuyan@caas.cn

**Keywords:** Eupelmidae, biological control, diapause, sensitive stage, storage

## Abstract

**Simple Summary:**

*Anastatus japonicus* Ashmead is an important egg parasitoid wasp and natural enemy of *Tessaratoma papillosa*. It is commercially used in biological control, and has effectively suppressed *T. papillosa* population for decades in China. In practice, medium temperature in spring always induces diapause in *A. japonicus*, leading to the delay of adult emergence and missing the best chance to control the pest. Accurately regulating the development of *A. japonicus* is a key technique for the production of biocontrol agents and field release applications. In this study, responsible environmental factors for the induction of diapause in *A. japonicus* were investigated. A short photoperiod and medium temperature led to diapause in *A. japonicus*. Second–third instar larva are the most sensitive stages to diapause stimuli. Diapausing mature larvae had a significantly higher survival rate after 180 days storage at 10 °C than that of nondiapausing mature larvae. Taken together, results suggest methods that could be exploited in the developmental regulation, field-release pretreatment technology, and long-term storage of *A. japonicus*.

**Abstract:**

*Anastatus japonicus* Ashmead is a widely used biological control agent against stink bugs that can be successfully reared using the large eggs of the Chinese silkworm. In this study, environmental factors responsible for the induction of diapause in *A. japonicus* were investigated on host eggs of the Chinese silkworm. *A. japonicus* exhibited a facultative, mature larval diapause within its host eggs. Second–third instar larva are the most sensitive stages to diapause stimuli. The accumulation of diapause stimuli during all the larval stages maximized the diapause response. A short photoperiod of 10L:14D and temperature of 17 °C led to the occurrence of the highest diapause response, while a long photoperiod (14L:10D) and low temperatures (11 and 14 °C) prevented the diapause. A specific exposure period was required to reach high diapause incidence. Diapausing mature larvae had a significantly higher survival rate after 180 days storage at 10 °C than that of nondiapausing mature larvae. Taken together, results suggest methods that could be exploited in the developmental regulation, field-release pretreatment technology, and long-term storage of *A. japonicus*.

## 1. Introduction

The litchi stinkbug (*Tessaratoma papillosa* Drury) is one of the most serious and destructive pests of litchi (*Litchi chinensis* Sonn) and longan (*Dimocarpus longana* Lour) in Southeast Asia [1,2,3,4]. *Anastatus japonicus* Ashmead (Hymenoptera: Eupelmidae) is an important egg parasitoid wasp and natural enemy of *T. papillosa* [5]. It is commercially used in biological control, and has effectively suppressed *T. papillosa* population for decades in China [6], where *A. japonicus* is mass-reared inside the eggs of *Antheraea pernyi* Guerin-Meneville (Lepidoptera: Saturniidae) [5,7]. In practice, medium temperature in spring always induces diapause in *A. japonicus*, leading to the delay of adult emergence and missing the best chance to control the pest, thus greatly compromising its control effect [6]. Accurately regulating the development of *A. japonicus* is a key technique for the production of biocontrol agents and field-release applications. Diapause is an adaptive strategy for insects to survive adverse seasonal environmental conditions [8,9,10,11]. Photoperiod and temperature are the primary factors regulating diapause among most insects, but other factors, such as host and nutritional status, may also have an effect [12,13,14,15,16].

In southern China, *A. japonicus* has eight generations per year with a life cycle of 23–33 days. Its life cycle passes through four stages: egg, larva, pupa, and adult. The egg, larva, and pupa develop inside the host eggs, and the adult leaves the host egg through a small emergence hole [5,17]. *A. japonicus* overwinter as mature larvae, and adults emerge in mid- to late March the following year. Peak oviposition by *T. papillosa* occurs from late March to May, and then gradually declines before ceasing by the end of August [18]; the rate of parasitism by *A. japonicus* in the field is very low before mid-May [18,19]. Thus, it is necessary to release enough *A. japonicus* before peak oviposition by *T. papillosa* to realize the effective control of *T. papillosa*.

Several studies on the bioecology of *Anastatus* reported the existence of a diapause or hibernation of the species in the field. For example, 97.6% of *Anastatus bifasciatus* Geoffroy prepupae could enter into diapause when average day lengths are 13.3 h and mean daily temperature is 23.2 °C [20]. *A. ramakrishnai* Mani can also enter into diapause in winter, and *A. umae* Boucek diapauses as pupa [21,22], but little is known about the environmental regulation of diapause. In our previous work, a temperature of 17 °C and photoperiod of 6L:18D induced 60% of *A. japonicus* into diapause, but how these environmental factors regulate diapause is still unclear [6]. Therefore, the main purpose of this research was to further explore environmental factors in charge of diapause induction in *A. japonicus*.

The sensitive stages that perceive diapause-inducing environmental signals are species-specific. They often occur in the diapause and preceding developmental stages [23]. The determination of the sensitive stage enables us to understand how *A. japonicus* perceives the environment cues that lead to diapause response. Therefore, another goal of this study was to determine the most sensitive stages to diapause-inducing signals. Inducing the initial stage also has an effect on diapause induction in many insects, such as *Chrysopa Formosa* Brauer, *Aphidius ervi* Haliday, *Laodelphax striatellus* Fallen, and *Chrysopa nigricornis* Burm [24,25,26,27,28].

The storage of natural enemies is a way to ensure their sufficient number at the time of release in biological control programs. At present, cold storage is the most commonly used method for the preservation of natural enemies (e.g., parasitoids) when demand for them is reduced [5,29,30]. However, with increasing storage time, the overall performance of the parasitoids worsens. Our previous results showed that the eclosion rate of *Anastatus fulloi* decreased to 47.49% after 12 months of storage at 10–15 °C [31]. After storage for three weeks at low temperatures, there was an abrupt decrease in the survival rate in *T. dendrolimi* [32]. Thus, the survival rate of *A. japonicus* at chilling temperatures is also investigated in the current study.

In the present study, the diapause induction of *A. japonicus* was investigated under laboratory conditions. Our experiments first investigated the effect of photoperiod, temperature, and exposure period on the diapause induction in *A. japonicus*. We then explored the most sensitive developmental stages to diapause-inducing conditions. Effects of the chilling period on the survival rate of *A. japonicus* were also investigated. Results presented suggest techniques that could be exploited in the developmental regulation, field-release pretreatment technology, and long-term storage of *A. japonicus*.

## 2. Materials and Methods

### 2.1. Insect Rearing and Experimental Conditions

*Anastatus japonicus* used in this study were obtained from MARA CHINA-CABI Joint Laboratory for Bio-Safety, Institute of Plant Protection, Chinese Academy of Agricultural Sciences, Beijing, China. The initial colony was initiated from field-parasitized brown marmorated stink bug (*Halyomorpha halys* Stal) eggs collected from the suburbs in Beijing. The colony of *A. japonicus* populations was maintained by using the eggs of *Antheraea pernyi* at 24 °C and 16L:8D in the laboratory for 1 year. Newly emerged *A. japonicus* were fed on 10% honey and water solution in a plastic box (32 cm in length, 25 cm in width, and 9 cm in height) with a lid at 24 °C and 16L:8D. A rectangular hole was produced in the middle of the lid and covered with a stainless-steel mesh screen (120 mesh). *Antheraea pernyi* eggs were used as the alternative host; the eggs were unfertilized and could not hatch. After 2 days of parasitization, *A. pernyi* eggs parasitized by *A. japonicus* were transferred into a new plastic box, and reared at 24 °C and 16L:8D before use. Eggs and larvae in the plastic box were exposed to different photoperiods and temperatures for observing diapause induction. Experiments were performed in climate chambers (Ningbo Dongnan, China) in which the relative humidity was set at 70%.

### 2.2. Effect of Temperature and Exposure Period on Diapause Induction 

To investigate the effect of temperature and exposure period on the induction of diapause, 2nd–3rd instar larvae of *A. japonicus* were incubated at 11 °C, 14 °C, 17 °C and 20 °C, respectively, and a photoperiod of 6L:18D. The developmental stage of *A. japonicus* was determined by dissecting *A. pernyi* eggs. After 2 days of parasitization, eggs of *A. pernyi* parasitized by *A. japonicus* were first incubated at 24 °C and 16L:8D for development. Larvae were transferred to diapause inducing conditions (11 °C, 14 °C, 17 °C and 20 °C, with photoperiods of 6L:18D) after all had entered into 2nd–3rd instar larvae. It is reported that *A. japonicus* could enter into diapause as mature larva [6]. Therefore, when all the larvae developed into mature larvae, they were transferred from the diapause inducing conditions (11 °C, 14 °C, 17 °C and 20 °C, 6L:18D) to development condition (24 °C, 16L:8D). After 12 days, *A. pernyi* eggs were dissected, and the developmental stage and survival of mature larvae were recorded. Each mature larva that had not pupated within 12 days at 24 °C, 16L: 8D was considered to be in diapause [6]. Incidence of diapause was calculated in each treatment. Approximately 50 individuals were examined for each treatment and each treatment was repeated three times.

In order to save the cost, full darkness or especially short photoperiod is often used in the mass rearing of *A. japonicus*. Therefore, we set this short photoperiod according to the needs of mass rearing of *A. japonicus*.

### 2.3. Effect of Photoperiod and Temperature on the Induction of Diapause

To investigate the effect of photoperiod and temperature on diapause induction, 2nd–3rd instar larvae of *A. japonicus* were reared at 14 and 17 °C under the varying photoperiods of 6L:18D, 10L:14D, and 14L:10D. When all the larvae had developed into mature larvae, they were transferred from the diapause-inducing condition to the development condition (24 °C, 16L:8D). After 12 days, *A. pernyi* eggs were dissected to record the number of diapausing individuals. Incidence of diapause was calculated as described above. Approximately 50 individuals were examined for each treatment, and each treatment was repeated three times.

### 2.4. Effect of Inducing Initial Stage on Diapause Induction

To investigate the effect of inducing initial stage on diapause induction, different stages (egg, 2nd–3rd instar, and mature larvae) of *A. japonicus* were incubated at 17 °C and under a photoperiod of 6L:18D for 45 days. Then, they were transferred from the diapause-inducing condition to the development condition (24 °C, 16L:8D). Incidence of diapause was calculated as described above. The culturing method was identical to that described above. Approximately 50 individuals were examined for each treatment, and each treatment was repeated three times.

### 2.5. Sensitive Stage to Diapause Induction

There are three larval instars in *Anastatus japonicus*. Our previous work showed that it enters diapause as a mature larva [6]. The host eggs parasitized by *A. japonicus* were divided into nine groups for different treatments. Individuals of *A. japonicus* at the beginning of different stages (egg, 1st–2nd instar, 2nd–3rd instar, and mature larva), were transferred either from diapause-preventing conditions (L: 24 °C, 16L:8D) to diapause-inducing conditions (S: 17 °C, 6L:18D) or vice versa according to the experimental protocols described in Table 1 [28]. The culturing method was identical to that described above. Incidence of diapause was calculated as described above. Approximately 50 individuals were examined for each treatment, and each treatment was repeated three times.

### 2.6. Effect of Chilling Period on Survival of Diapausing and Nondiapausing Mature Larvae of A. japonicus

To investigate the effect of chilling on survival rate, diapausing mature larvae induced under 10L:14D at 17 °C for 45 days were placed at 10 °C for 0 and 180 days in continuous darkness. Nondiapausing mature larvae were reared at 24 °C and 16L:8D, and placed at 10 °C for 0 and 180 days in continuous darkness. After chilling, mature larvae were transferred to 24 °C and 16L:8D until adult emergence. The percentage of adult emergence was calculated for each treatment. Approximately 100 individuals were examined for each treatment, and each treatment was repeated three times.

### 2.7. Statistical Analyses

**Figure 1:**
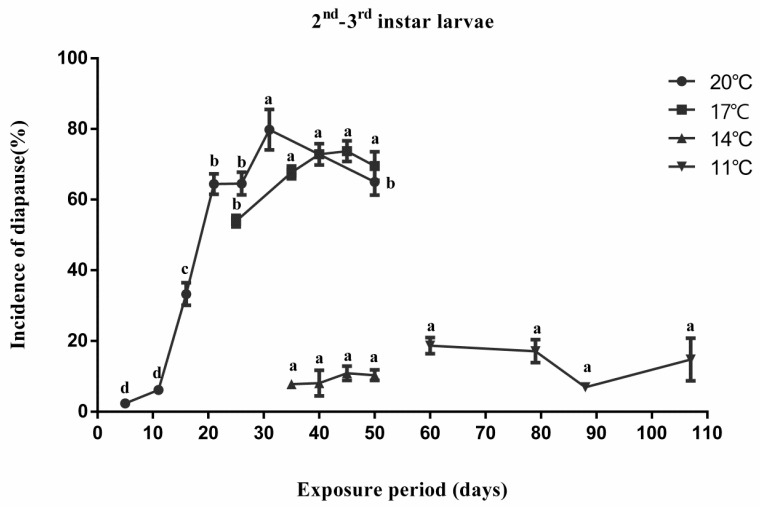
Effect of temperature and exposure period on diapause induction in *Anastatus japonicus* when 2nd–3rd instar larvae used as inducing initial stage (mean ± SE%). Different letters indicate significant difference (one-way ANOVA, followed by LSD multiple-comparison tests, *p* < 0.05) between different exposure periods at the same temperature.

In Section 2.2 and Section 2.4, the effect of temperature, exposure period, and inducing initial stage on diapause induction was assessed using one-way ANOVA followed by LSD multiple-comparison tests. Dataset normality was checked by using the Shapiro–Wilk test before analysis. In Section 2.3, the impact of temperature and photoperiod, and the interaction of these two factors on diapause was assessed using two-way ANOVA, followed by Bonferroni’s multiple-comparison tests. The same statistical analysis method was used in Section 2.6. Diapause incidences and eclosion rates were square-root-transformed, whereas means and standard error of untransformed data are shown in Figure 1.

Statistical analyses were carried out using SPSS 21 (IBM Inc., Armonk, NY, USA) statistical software. The figures were created by GraphPad Prism 6 (GraphPad).

## 3. Results

### 3.1. Effect of Temperature and Exposure Period on Diapause Induction 

Diapause induction in *A. japonicus* was affected by temperature and exposure period under a photoperiod of 6L:18D (Figure 1). More than 80% development proceeded at 11 and 14 °C regardless of exposure period in the diapause-inducing condition (11 and 14 °C, 6L:18D). *A. japonicus* was induced into diapause at temperatures of 17 and 20 °C under the photoperiod of 6L:18D. Diapause incidence significantly increased as exposure period was prolonged in 17 °C (*F*_(4,14)_ = 7.256, *p* = 0.005) and 20 °C (*F*_(6,20)_ = 91.771, *p* < 0.001). A high incidence of diapause was observed at an exposure period of 30–50 days at 17 or 20 °C.

All treatments were transferred from diapause-inducing conditions to development conditions after reaching the mature larval stage. A different exposure time was needed to reach mature larvae at different temperatures. Therefore, the start and end times were different in this setup.

### 3.2. Effect of Photoperiod and Temperature on Diapause Induction 

**Figure 2:**
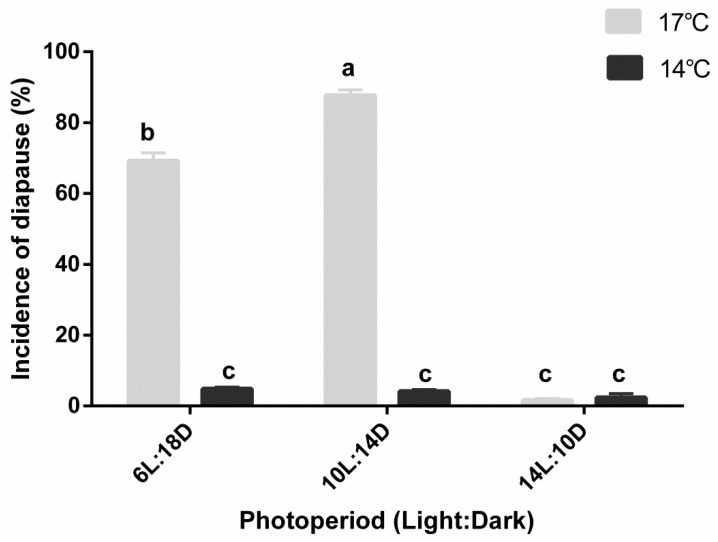
Effect of photoperiod on diapause induction in *A. japonicus* at different temperatures (mean ± SE%). Different letters indicate significant difference (two-way ANOVA, followed by Bonferroni’s multiple-comparison tests, *p* < 0.05) between different temperatures and photoperiods. Photoperiod, temperature, and their interactions all significantly influenced diapause induction in *A. japonicus* (temperature effect: *F*_(1,37)_ = 1018.878, *p* < 0.001; photoperiod effect: *F*_(2,37)_ = 330.119, *p* < 0.001; temperature × photoperiod interactions: *F*_(2,37)_ = 273.080, *p* < 0.001).

The effect of photoperiod on diapause induction in *A. japonicus* at different temperatures is shown in Figure 2. The photoperiodic response bar chart showed a typical long-day response type. Incidence of diapause increased significantly with shortening photophase at 17 °C. More than 98% individuals directly developed at a day length of 14 h, whereas more than 85% and 65% larvae entered diapause at 10L:14D and 6L:18D, respectively. Thus, a photoperiod of 10L:14D could induce more *A. japonicus* into diapause than the photoperiods of 6L:18D and 14L:10D at 17 °C could when the same stage was used as the initial inducing stage. However, a low temperature of 14 °C nearly abrogated the diapause-inducing effects of a short day, and more than 95% development proceeded at 14 °C regardless of photoperiods. The day length of 14 h nearly abrogated the diapause-inducing effects of a medium temperature (17 °C), and more than 95% development proceeded at day lengths of 14 h regardless of temperature.

Photoperiod, temperature, and their interactions all significantly influenced the induction of diapause in *A. japonicus* (temperature effect: *F*_(1,37)_ = 1018.878, *p* < 0.001; photoperiod effect: *F*_(2,37)_ = 330.119, *p* < 0.001; temperature × photoperiod interactions: *F*_(2,37)_ = 273.080, *p* < 0.001). A short photoperiod of 10L:14D and temperature of 17 °C led to the occurrence of the highest diapause response, while a long photoperiod (14L:10D) and low temperature (14 °C) prevented the diapause.

### 3.3. Effect of Inducing Initial Stage on Diapause Induction

**Figure 3:**
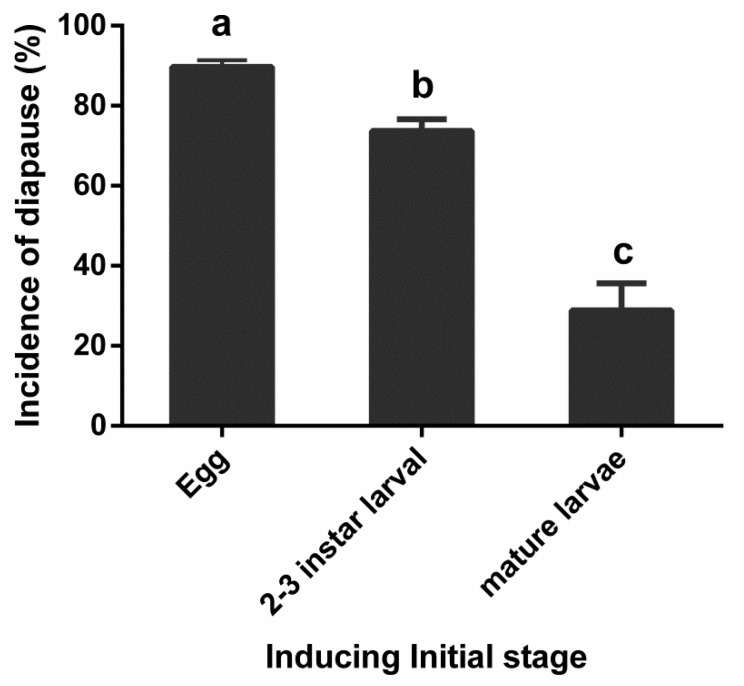
Effect of initial inducing stage on diapause induction of *Anastatus japonicus* at 17 °C under photoperiods of 6L:18D (mean ± SE%). Different letters indicate significant difference (one-way ANOVA, followed by LSD multiple-comparison tests, *p* < 0.05) between different initial inducing stages.

To investigate the effect of the initial inducing stage on diapause induction, different stages (eggs, 2nd–3rd instar, and mature larvae) of *A. japonicus* were incubated at 17 °C under photoperiods of 6L:18D (Figure 3). Initial inducing stage significantly influenced the induction of diapause in *A. japonicus* (*F*_(2,6)_ = 50.028, *p* < 0.001). Significantly higher incidences of diapause were induced when eggs were used as the initial inducing stage than when 2nd–3rd instar or mature larvae were used (Figure 3). When mature larvae were used as the initial inducing stage, the incidence of diapause significantly decreased to less than 35%.

### 3.4. Sensitive Stage to Diapause Induction

The incidence of mature larval diapause in *A. japonicus* was largely decided by the conditions experienced by 2nd–3rd instar larvae (Table 1). Most mature larvae entered diapause when the 2nd–3rd instar larvae were exposed to 17 °C and 6L:18D (Table 1c). Exposure of only the eggs or 1st–2nd instar larvae to 17 °C and 6L:18D resulted in a decrease in the incidence of diapause (<10%) (Table 1a,b), but higher incidences of diapause were induced if earlier instar larvae or mature larvae and 2nd–3rd instar larvae were exposed to 17 °C and 6L:18D (Table 1e–i). The induction of diapause in mature larvae when 2nd–3rd instar (Table 1c) or mature (Table 1d) larvae were exposed to diapause-inducing conditions indicates that these stages were sensitive to diapause stimuli. However, a higher incidence of diapause was observed when 2nd–3rd instar larvae were exposed than with the mature larvae. This indicates that 2nd–3rd instar larvae were the most sensitive stages to diapause stimuli.

### 3.5. Effect of Chilling Period on Survival of Diapausing and Nondiapausing Mature Larvae of A. japonicus

**Figure 4:**
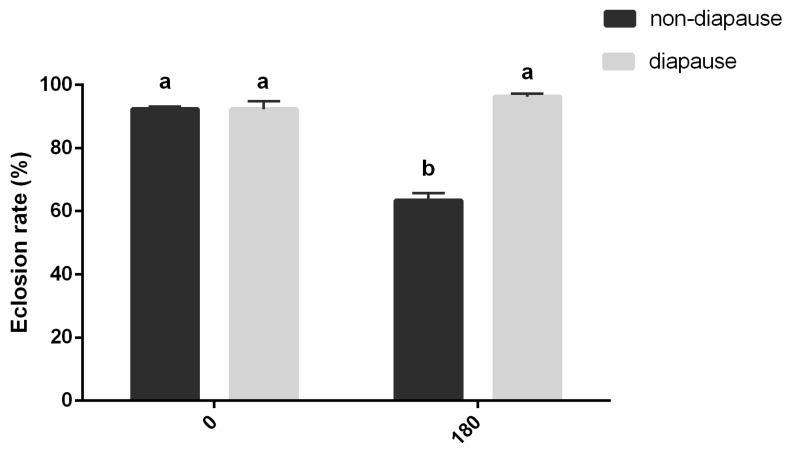
Effect of chilling period on survival of diapausing and nondiapausing mature larvae of *A. japonicus* (mean ± SE%). Different letters indicate significant difference (two-way ANOVA followed by Bonferroni’s multiple comparison tests, *p* < 0.05) between different chilling periods and diapause or not. Chilling period, diapause, and their interactions all significantly influenced eclosion rate in *A. japonicus* (diapause effect: *F*_(1,20)_ = 54.069, *p* < 0.001; chilling period effect: *F*_(1,20)_ = 19.278, *p* < 0.001; diapause × chilling period interactions: *F*_(1,20)_ = 45.637, *p* < 0.001).

To investigate the effect of chilling on survival rate, diapausing and nondiapausing mature larvae were placed at 10 °C for 180 days in continuous darkness (Figure 4). Chilling period, diapause, and their interactions all significantly influenced the eclosion rate in *A. japonicus* (diapause effect: *F*_(1,20)_ = 54.069, *p* < 0.001; chilling period effect: *F*_(1,20)_ = 19.278, *p* < 0.001; diapause × chilling period interactions: *F*_(1,20)_ = 45.637, *p* < 0.001). Diapausing mature larvae of *A. japonicus* had a significantly higher eclosion rate after storage in 10 °C for 180 days than that of nondiapausing mature larvae. The eclosion rate of diapausing mature larvae storage for 180 days is similar with nondiapausing and diapausing mature larvae without chilling treatment. Adults that had emerged after treatment had normal mating and oviposition action, though fecundity was not recorded.

## 4. Discussion

The storage of *Anastatus* parasitoids ensures their availability in sufficient numbers at the time of release. So far, storage in low temperatures has been the primary method to achieve a longer storage time for *Anastatus* [5]. Our results showed that diapausing mature larvae of *A. japonicus* have a higher survival rate after storage in 10 °C than that of nondiapausing mature larvae. This also coincides with the conclusion in the literature that parasitizing host eggs with diapause-induced parasitoids leads to high eclosion incidence. Laing and Corrigan found that the emergence rate of diapausing *Trichogramma minutum* Riley was >50% after 300 days of storage [33]. Similarly, the eclosion incidence of *Trichogramma dendrolimi* Matsumura was around 80% after storage at low temperatures for 3 months [16]. In this study, the eclosion proportion of *A. japonicus* was more than 90% after 180 days of storage at 10 °C. The present study suggested that the induction of diapause could be an effective way to achieve the long-term storage of *Anastatus* parasitoids. The life span and fecundity of adults that emerged after storage in 10 °C need to be further studied.

The sensitive stage usually occurs in the diapause or preceding developmental stages [14]. Our observations indicated that 2nd–3rd instar larvae, which immediately precede the diapause stage, are the most sensitive to diapause-inducing signals. Although eggs and 1st–2nd instar larvae were insensitive to diapause-inducing stimuli, a short photoperiod and medium temperature elevated the incidence of diapause in young larval stages and/or mature larvae of *A. japonicus*, suggesting that sensitivity was not limited to 2nd–3rd instar larvae. Similar responses were recorded in many other insect species [24,25,26,27,28]. Therefore, diapause stimuli should be carried out during all larval stages to maximize diapause response. Results of this study show that the pupae or adults of *A. japonicus* that are not sensitive to diapause-inducing conditions in the field could be released in medium temperatures in spring that often induce diapause. These field-released *A. japonicus* can control associated pests in time to enhance the effect of biological control.

Photoperiod, temperature, and their interactions significantly influence the induction of diapause in *A. japonicus,* and, like other *Anastatus* species [20,21,22], short photoperiods (6L:18D and 10L:14D) and medium temperatures (17 and 20 °C) can induce diapause. In this study, a low temperature of 14 °C nearly abrogated the diapause-inducing effects of a short photoperiod, while the long photoperiod with 14 °C nearly abrogated the diapause-inducing effects of temperature (17 °C). These results suggest that diapause induction in *A. japonicus* requires a combination of specific temperature and photoperiod conditions. The reasonable explanation for this result may be that, when exposed to short photoperiods with a moderate temperature of 17 °C, *A. japonicus* could slowly develop and prepare for diapause by accumulating energy and antifreeze substances, and secreting hormones. However, when exposed to short days with a low temperature of 11 and 14 °C, the metabolic rate of *A. japonicus* is abnormally slow, and it is difficult for it to accumulate enough energy and secrete sufficient hormones to complete diapause preparation, so it cannot enter diapause. These speculated physiological changes caused by lower temperature need further molecular testing. This phenomenon is very common among other parasitoid wasps, including *Aphidius gifuensis* Ashmead [23], *Microplitis mediator* (Haliday) [34], and *Coeloides brunneri* Viereck [35]. A long photoperiod is usually a signal that a favorable environment is coming, so *A. japonicus* does not enter diapause, which is an adaptive strategy for insects to survive adverse seasonal environmental conditions. The highest diapause response occurred under a combination of a short photoperiod of 10L:14D and a temperature of 17 °C.

There is negative correlation between the proportion of hibernation in *A. japonicus* and temperature. When the temperature is lower, the incidence of hibernation is higher in *A. japonicus* [17]. However, in our study, the incidence of diapause in *A. japonicus* was higher at temperatures of 17 and 20 °C than that at 11 and 14 °C. This indicates the existence of different mechanisms between diapause and hibernation in *Anastatus*.

This study indicates that diapause likely occurs in mid-October in northern China, since the natural photoperiod decreases to 10.5 h by late October, and the daily average temperature drops to 19 °C by mid-October. In Beijing, *Anastatus*-parasitized *Lycorma delicatula* White eggs overwintered as mature larvae inside the host eggs and emerged in late March to early April; only about 15% larvae entered into diapause and emerged in August and September [36]. This indicates that *Anastatus*-parasitized *Lycorma delicatula* White eggs can stay in diapause for 1 year under field conditions in Beijing. In Switzerland, eggs parasitized by *A. bifasciatus* between early August and mid-September were able to overwinter. However, eggs parasitized after mid-September could not reach the final instar necessary for successful overwintering [20].

Although a clear eclosion peak of adults occurred 4 weeks after parasitism, the duration of the emergence of *A. japonicus* was extremely prolonged over a period of one month. This eclosion pattern is known as “risk spreading” (bet hedging), which may have evolved in deal with changeable circumstances [37]. However, the duration of emergence is shortened to 2–3 weeks in larvae that went through diapause (unpublished data), which contributes to the accumulation of a large population size in a short time in the mass rearing of parasitoid wasps.

A combination of environmental and endogenous factors is usually involved in the complex process of diapause termination [9,28,38]. Increasing studies prove that a low temperature is not a prerequisite for diapause termination in many insect species, and some insects need to complete the diapause progress at medium or high temperatures [38]. In insect species such as *Trichogramma dendrolimi* Matsumura, *Chrysopa Formosa* Brauer, *Sitodiplosis mosellana* Gehin, and *Listronotus maculicollis* Kirby, exposure to chilling temperatures is necessary for the completion of diapause [28,38,39,40,41,42]. However, in our study, diapause in the mature larvae of *A. japonicus* was terminated under a high temperature (24 °C) and long photoperiod (16L:8D) (Figure 4). The incidence of diapause termination can reach 92.26% if the successful emergence of mature larvae is regarded as diapause termination (Figure 4).

## 5. Conclusions

In summary, the present study demonstrated that the induction of diapause in *A. japonicus* is governed by photoperiod and temperature. Mature larval diapause did not occur unless 2nd–3rd instar or mature larvae received the right levels of short-day photoperiodic and medium-temperature signals. To maximize the diapause response, the accumulation of diapause stimuli during all larval stages was needed. Additionally, a specific exposure period was required to reach a high diapause incidence. To the best of our knowledge, diapause induction in *A. japonicus* under laboratory conditions has not yet been reported. Diapausing mature larvae had a higher survival rate after long-term storage than that of nondiapausing mature larvae. Taken together, results suggest methods that could be exploited in the developmental regulation, field-release pretreatment technology, and long-term storage of *A. japonicus*. The diapause termination, postdiapause developmental features, and the molecular mechanism of diapause in *A. japonicus* need to be further studied.

## Figures and Tables

**Table 1:** Incidence of diapause of mature larvae after culturing *Anastatus japonicus* individuals under diapause-preventing condition (L: 24 °C, 16L:8D) and transferring them to diapause-inducing condition (S: 17 °C, 6L:18D) at different stages.

Groups	Egg	1st–2nd Instar Larvae	2nd–3rd Instar Larvae	Matur Larvae	Diapause
a	S	L	L	L	4.41%
b	L	S	L	L	7.50%
c	L	L	S	L	56.13%
d	L	L	L	S	32.17%
e	L	L	S	S	61.81%
f	L	S	S	L	84.68%
g	S	S	S	L	90%
h	S	L	S	L	65.32%
i	S	S	L	L	26.49%
